# Early Subclinical Atherosclerosis in Gestational Diabetes: The Predictive Role of Routine Biomarkers and Nutrigenetic Variants

**DOI:** 10.1155/2018/9242579

**Published:** 2018-12-24

**Authors:** Marica Franzago, Federica Fraticelli, Marta Di Nicola, Francesco Bianco, Daniela Marchetti, Claudio Celentano, Marco Liberati, Raffaele De Caterina, Liborio Stuppia, Ester Vitacolonna

**Affiliations:** ^1^Department of Medicine and Aging, School of Medicine and Health Sciences, “G. d'Annunzio” University, Chieti-Pescara, Chieti, Italy; ^2^Laboratory of Biostatistics, Department of Medical, Oral and Biotechnological Sciences, “G. d'Annunzio” University, Chieti-Pescara, Chieti, Italy; ^3^Institute of Cardiology, School of Medicine and Health Sciences, “G. d'Annunzio” University, Chieti-Pescara, Chieti, Italy; ^4^Department of Psychological, Health and Territorial Sciences, School of Medicine and Health Sciences, “G. d'Annunzio” University, Chieti-Pescara, Chieti, Italy

## Abstract

Gestational diabetes mellitus (GDM) can be considered a silent risk for out-of-pregnancy diabetes mellitus (DM) and cardiovascular disease (CVD) later in life. We aimed to assess the predictive role of 3^rd^ trimester lipid profile during pregnancy for the susceptibility to markers of subclinical atherosclerosis (CVD susceptibility) at 3 years in a cohort of women with history of GDM. A secondary aim is to evaluate the usefulness of novel nutrigenetic markers, in addition to traditional parameters, for predicting early subclinical atherosclerosis in such women in order to plan adequate early prevention interventions. We assessed 28 consecutive GDM women in whom we collected socio-demographic characteristics and clinical and anthropometric parameters at the 3^rd^ trimester of pregnancy. In a single blood sample, from each patient, we assessed 9 single nucleotide polymorphisms (SNPs) from 9 genes related to nutrients and metabolism, which were genotyped by High Resolution Melting analysis. All women then attended a 3-year-postpartum follow-up and on that occasion performed an oral glucose tolerance test (OGTT, with 75 g oral glucose), the measurement of carotid artery intima-media thickness (cIMT), and analyses of metabolic parameters. In addition, we evaluated the physical activity level and the adherence to Mediterranean diet (MedDiet) using the International Physical Activity Questionnaire (IPAQ-*short version*) and PREDIMED questionnaires. We found an association between 3^rd^ trimester triglycerides and cIMT (*p* = 0.014). We also found significant associations between the APOA5 CC genotype and cIMT after adjustments for age and body mass index (*p* = 0.045) and between the interaction CC APOA5/CC LDLR and cIMT (*p* = 0.010). At the follow-up, the cohort also featured a mean BMI in the overweight range and a high mean waist circumference. We found no difference in the MedDiet adherence, physical activity, and smoking but an inverse correlation between the PREDIMED and the IPAQ scores with the IMT. In conclusion, this preliminary study provides insight into the predictive role of lipid profile during pregnancy and of some genetic variants on cIMT taken as a parameter of subclinical CVD susceptibility in GDM.

## 1. Introduction

Gestational diabetes mellitus (GDM) is defined as “diabetes diagnosed in the 2^nd^ or 3^rd^ trimester of pregnancy, with or without remission after end of pregnancy” [1]. GDM prevalence has been reported to vary between 1% and 28% [2] and is increasing, especially in developed countries [3]. GDM may have clinical implications for both maternal and fetal adverse outcomes and for the later development of type 2 diabetes in the years following pregnancy. In addition to type 2 diabetes, women with GDM are also at greater risk of overt cardiovascular disease (CVD) later in life. Several modifiable and unmodifiable risk factors are involved in the connection between GDM and subsequent CVD: these include hyperglycemia and impaired glucose tolerance, atherogenic lipid profiles, higher age, and elevated high-sensitivity C-reactive protein (CRP) [4]. Particularly, the development of diabetes purports an increased risk of developing later CVD [5, 6]. Nevertheless, mechanisms linking GDM and CVD are still unclear [6–11]. Metabolic impairments, including dyslipidemia and vascular dysfunction, are common later in life in women with previous GDM (pGDM) [12, 13]. In these women, elevated markers of inflammation, decreased levels of adiponectin, increased peripheral resistance, and decreased cardiac output have been detected [11]. Women with pGDM also have higher total cholesterol (TC), low-density lipoprotein cholesterol (LDL-C), and triglycerides (TG), as well as lower high-density lipoprotein cholesterol (HDL-C), compared with healthy women of the same age, suggesting that pGDM women have a greater susceptibility to be exposed to “an atherogenic insult” [14, 15]. Here, an obesogenic lifestyle (unhealthy diet and physical inactivity) and a genetic predisposition [16] likely explains the later development of CVD in most such women.

Carotid artery intima-media thickness (cIMT) is a subclinical measure of early atherosclerosis that strongly predicts heart disease and stroke, particularly in women [17, 18], and also predicts the development of CVD from GDM [4, 19].

Several studies recently carried out in women with pGDM have shown higher values of endothelial dysfunction markers and of cIMT in such women compared with controls, despite the absence of evident metabolic abnormalities [10, 19–21]. Previous studies of ours [22, 23] also showed a relationship between several nutrigenetic variants and cardiometabolic risk factors in women with or without GDM, suggesting the need to consider such factors in association with routinely assessed markers (such as lipid profile during pregnancy) for their role in the development of post-GDM CVD.

In this context, in the present study, we aimed to assess the joint predictive role of lipid profile during pregnancy and of some genetic variants on cIMT taken as a parameter of subclinical atherosclerosis and indicating an early susceptibility to CVD in a cohort of women with GDM history. If proven predictive, such prediction models would allow the planning of adequate primary cardiometabolic disease (CMD) prevention interventions in post-GDM women.

## 2. Materials and Methods

### 2.1. Study Design and Participants

Twenty-eight consecutive pGDM women attending the Diabetes and Metabolism Unit and the Obstetrics and Gynaecology Clinic, School of Medicine and Health Sciences, “G. d'Annunzio” University of Chieti-Hospital “SS Annunziata” of Chieti, were recruited. Socio-demographic characteristics and clinical parameters, such as blood glucose, TC, HDL-C, LDL-C, TG, and blood pressure, were collected. BMI was measured at the beginning (BMI 1) and at the end of pregnancy (BMI 2). A blood sample was obtained from each patient and nine single nucleotide polymorphisms (SNPs) from 9 genes related to nutrients and metabolism were included in the analysis.

All 28 women attended a 3-year-postpartum follow-up. Postpartum glucose tolerance (75 g oral glucose tolerance test (OGTT)) was assessed. Clinical parameters were collected in all subjects. Cardiovascular and metabolic markers were analyzed, including total, HDL, and LDL cholesterol levels; TG; homocysteine; and carotid artery IMT. In addition, HbA1C and fasting blood glucose were measured.

Adherence to the Mediterranean diet (MedDiet) was evaluated through a validated 14-item questionnaire (PREDIMED), which generates a range of possible scores namely (i) no adherence (score ≤ 5), (ii) medium adherence (6 ≤ score ≤ 9), and (iii) maximum adherence (score ≥ 10) [24]. In addition, physical activity (PA) was assessed using a *short version* of the International Physical Activity Questionnaire (IPAQ), comprising 7 items concerning PA, 4 relating to demographic information, and the remaining 6 about the comprehension of the questionnaire [25]. IPAQ registers three different levels of intensity (low, moderate, and high PA).

All participants gave their written informed consent prior to their inclusion in the study. The study was approved by the Ethics Committee of the “G. d'Annunzio” University, Chieti-Pescara, Italy.

### 2.2. Inclusion and Exclusion Criteria

The inclusion criteria admitted women with ≥18 years of age with pGDM. The GDM diagnosis was confirmed when established both at the 16–18th and the 24–28th weeks of gestation, according to the International Association of Diabetes and Pregnancy Study Groups (IADPSG) criteria [26].

The exclusion criteria were women with type 1 or 2 prepregnancy diabetes, overt diabetes, or monogenic diabetes, specifically GCK diabetes.

### 2.3. Gene and SNP Selection

The genetic analysis was conducted at the Laboratory of Molecular Genetics, School of Medicine and Health Sciences, “G. d'Annunzio” University of Chieti. A total of 9 SNPs previously associated in literature with obesity, lipid, and glucose metabolism were selected. These SNPs, on different loci, have been associated with cardiovascular disease in previous studies, under the assumption that these variants may also contribute to cardiovascular risk assessment [22, 23].

In particular, three of these variants, namely, rs7903146 (*C* > *T*) in *TCF7L2*, rs1801282 (*C* > *G*) in *PPARG2*, and rs8192678 (*C* > *T*) in *PPARGC1A* are involved in carbohydrate metabolism; other three, namely, rs662799 (*T* > *C*) in *APOA5*, rs2228671 (*C* > *T*) in *LDLR*, and rs1260326 (*C* > *T*) in *GCKR* are involved in fat metabolism. In addition, two other SNPs, rs9939609 (*T* > *A*) in *FTO* and rs17782313 (*T* > *C*) near *MC4R*, associated with hunger control, were also selected. Finally, rs1801133 (*C* > *T*) in *MTHFR*, involved in folate metabolism, was also genotyped. It is of great relevance that all the selected gene variants make up a panel of CVD markers, which could provide a unique opportunity to use genetic information in clinical practice to predict early CVD in pGDM women [22, 23].

All SNPs were genotyped by High Resolution Melting (HRM) analysis. HRM was performed on 96-well PikoReal Real-Time PCR System (Thermo Scientific™) using the Luminaris Color HRM Master Mix (Thermo Scientific™) according to the manufacturer's instructions, as previously described [22].

### 2.4. Carotid Intima-Media Thickness Assessment

Carotid intima-media thickness (cIMT) was assessed in the Institute of Cardiology, School of Medicine and Health Sciences, “G. d'Annunzio” University of Chieti.

Left and right intima-media thickness (IMT) was measured on common carotid posterior wall, 1 centimeter from bulb bifurcation on each side, according to the latest European Society of Cardiology (ESC) and European Society of Hypertension (ESH) guidelines [27].

All the exams were performed with a dedicated Esaote MyLab 30 Gold portable ultrasound, with a standard 7.5 MHz linear probe, provided with Quality Intima Media Thickness (QIMT™) software. The software uses radio frequency data processing signal in real time and ensures high accuracy and low intra- and interobserver variability [28]. One expert operator (FB) performed all the exams in a blinded fashion from genetics; before the automatic measurement of QIMT, a manual image acquisition of carotid vessels was obtained with each participant lying supine, with the neck hyperextended. At each patient was attributed the mean value between three measurements for QIMT. Concordance correlation coefficient between the three measurements was 0.99 (95% CI: 0.98-0.99).

### 2.5. Statistical Analysis

We estimated the minimum required sample size for the correlation analysis on the basis of previously observed data or published results. The minimum sample size (*n* = 28) was determined in order to obtain an expected correlation coefficient (*r* = 0.5) [20] between cIMT and lipid profile parameters with at least 80% of desired statistical power level and an alpha error rate of 5%.

The quantitative variables were summarized as mean and standard deviation (SD) or median and interquartile range (IQR), according to their distribution. Qualitative variables were summarized as frequency and percentage. Shapiro-Wilk's test was performed to evaluate the departures from normality distribution for each variable.

Lin's concordance correlation coefficient (CCC) was calculated along with the 95% confidence intervals of assessing the intraobserver reproducibility of measurements.

Nonparametric Kruskal-Wallis test was performed to test the effect of different APOA5 genotypes on levels of cIMT and on levels of TC, HDL-C, LDL-C, and TG.

The relationship between cIMT and TC, HDL-C, LDL-C, and TG at the 3^rd^ trimester was explored by linear multiple regression analysis adjusted for age and BMI. The univariate regressions between cIMT and lipid profile at the 3^rd^ trimester of pregnancy were reported graphically as a scattergram.

Hardy-Weinberg equilibrium (HWE) deviations in the genotype frequency distributions were calculated using the chi-square analysis.

The level of statistical significance was set at *p* < 0.05. Statistical analysis was performed using the Statistical Package for Social Science (SPSS) software for Windows (SPSS, Chicago, IL, USA) and Stata v14.1 (StataCorp, College Station, TX).

## 3. Results

The demographic and clinical characteristics of the cohort of women, both during pregnancy and at follow-up, are reported in Tables 1 and 2, respectively. A total of 28 women were included in the study. No difference has been found in the adherence to the MedDiet, PA, and smoking among the sample.

Although not statistically significant, an inverse correlation between the PREDIMED and the IPAQ scores with the cIMT values was found (Rho = −0.060, *p* = 0.768 and Rho = −0.276, *p* = 0.163, respectively). In addition, our results showed a connection between the women's waist circumference and cIMT values (Rho = 0.378, *p* = 0.057) at the follow-up, which, however, does not reach a statistical significance.

**Figure 1:**
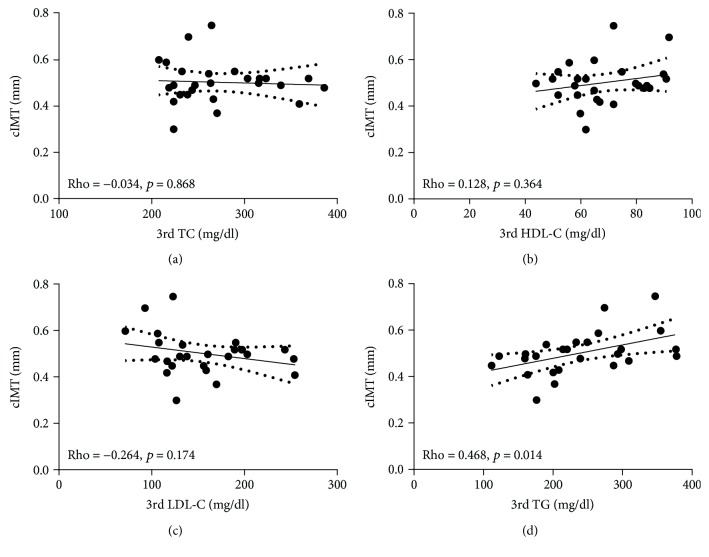
Nonparametric correlation analysis between cIMT and lipid profile during 3^rd^ trimester of pregnancy.

**Figure 2:**
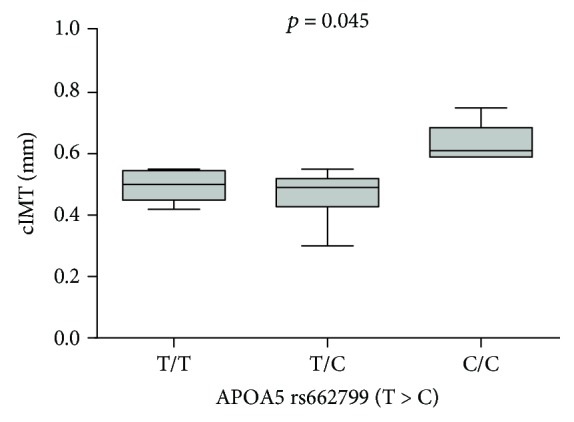
Box-whisker graphs of cIMT values with respect to APOA5 genotypes. Box-whisker plots show the 25^th^ and 75^th^ percentile range (box) with Tukey 95% confidence intervals (whiskers) and median values (transverse lines in the box). *p* value in figure is relative to Kruskal-Wallis test.

Furthermore, a significant positive relation between the 3^rd^ trimester TG and cIMT (Rho = 0.468; *p* = 0.014) was found (Figure 1). The genotype distribution of investigated SNPs in patients is reported in Table 3. All the investigated genotype frequencies were within the Hardy-Weinberg equilibrium range (*χ*^2^ test *p* value > 0.05). Also, a significant association was found in the codominant model (TT vs. TC vs. CC) between APOA5 CC genotype and cIMT after adjustments for age and BMI (0.50 ± 0.07 vs 0.48 ± 0.08 vs 0.65 ± 0.08; *p* = 0.045) (Figure 2). Finally, a significant association between the interaction CC APOA5/CC LDLR and cIMT (*p* = 0.010) has been observed.

No statistically significant differences were found among the 3^rd^ trimester lipid profile, PREDIMED, and IPAQ scores in different APOA5 genotypes.

No other significant differences were detected with respect to other genes.

## 4. Discussion

The main aim of the present study was to assess the joint predictive role of lipid profile during pregnancy and of some genetic variants, cIMT taken as a parameter of subclinical atherosclerosis, and indicating an early susceptibility to CVD in a cohort of women with GDM history. Another aim was to examine new nutrigenetic markers as well as traditional parameters to predict early subclinical atherosclerosis in pGDM and to plan adequate early prevention interventions.

Several routine parameters and biochemical markers are currently available to quantify the risk in pGDM women to develop diabetes after pregnancy [3]. Although the incidence of CVD events in young women is low, an early identification of possible CMD risk may provide an irreplaceable opportunity for well-timed intervention and timely prevention. In order to reduce not only the occurrence of diabetes but also its subsequent cardiovascular complications, it is necessary to improve the provision of postpartum follow-ups. In fact, many authors have highlighted the close relationship between common CVD risk factors and a GDM history. In addition to traditional parameters, cIMT and arterial stiffness (RFQAS) values can be significant in assessing the risk for heart disease and strokes [18]. Bo et al. [20] measured cIMT in 82 women with a history of GDM and 113 without one, 6.5 years after delivery: their study showed that women with pGDM, regardless of their BMI and the presence of metabolic abnormalities, displayed remarkably higher E-selectin, ICAM-1, and IMT values than controls. IMT proved to be significantly associated with pGDM in a regression model, after adjustments for BMI, waist circumference, blood pressure, and glucose values. Volpe et al. [21] measured cIMT in 28 women with and 24 without a history of GDM 2 years after delivery, finding that young women with pGDM presented early signs of vessel involvement, albeit within upper normal levels. GDM and control groups differed in terms of their main metabolic syndrome components, such as waist circumference, blood pressure, fasting plasma glucose, and TG, all significantly higher in GDM women than in the control group.

Kaul et al. [29] observed that GDM was associated with 1.4 times higher rates of CVD. Then, Retnakaran and Shah [11] confirmed similar results, noting that women with GDM have an elevated risk of bad cardiovascular outcomes, even in the absence of type 2 diabetes.

Hypertriglyceridemia and low HDL-C are known to be characteristic traits of type 2 diabetes. It may not be surprising that in our previous studies their presence was detected in women with pGDM. Although the CV risk implications of hypertriglyceridemia and low HDL-C remain controversial, the CV significance of LDL-C and its main lipoprotein (apolipoprotein B (apoB)) is well established [30]. In our previous studies, GDM women showed significantly higher serum concentrations of TC and LDL-C during the 3^rd^ trimester than the control group, and a significant correlation was observed between lipid parameters and some polymorphisms in genes APOA5 and LDLR; also, TG were higher in GDM women than controls, although not reaching statistical significance [23]. Interestingly, in our present study, an association between 3^rd^ trimester TG and cIMT has been found (*p* = 0.014).

These remarkable results echo those of Di Cianni et al. [31], who reported that TC, TG, LDL-C, glucose, and systolic blood pressure were all significantly higher among the GDM cohort, suggesting a condition similar to the metabolic syndrome occurring in these women. These peculiar changes in lipoprotein profile may favour endothelial damage in pregnancy [32, 33]. The GDM lipid profile is very similar to the one accompanying the insulin resistance in the metabolic syndrome. More recently, Gongora and Wenger [34] showed that women with GDM have a more atherogenic lipid profile by three months postpartum, characterized by an increase of cIMT compared to controls and that the risk of developing metabolic syndrome increased by up to 10% in those with pGDM.

It is known that pregnancy is a stress test, as hyperglycemia seems to have a significant impact on the CV system during this limited period [35]; we could hypothesize that lipid pattern modifications during pregnancy with GDM are an injury which results in a possible susceptibility to future CVD risk. Unfortunately, the mechanisms involved in an increased risk of CVD in pGDM need to be further investigated. GDM women's lipid profile displays a preponderance of small dense LDL particles; also, they present an increased susceptibility of LDL oxidation during pregnancy [30].

These data reveal the necessity of monitoring women with GDM adequately and long term, to prevent both CVD and diabetes risks. Unfortunately, several studies showed low rates of postdelivery glucose testing [36, 37]. It should be emphasised that, to date, the postpartum screening of women with pGDM is still suboptimal [3]; therefore, this issue also prompts the need to identify a practical and feasible tool which ideally should include panel genes and routine clinical and metabolic parameters, to identify GDM women at high risk of diabetes and CVD [23] and summon them for follow-ups. Previous investigations highlighted that women with pGDM have an increased risk of CVD later in life as a result of a combination of genetic factors and gene-diet interaction.

Furthermore, it is essential to define the characteristics of the studied gene variants. Interestingly, a previous Italian study showed that APOA5-1131T > C may affect the risk of early-onset myocardial infarction (MI), with an odds ratio of 1.44 (CI: 1.23–1.69) per C allele [38]. Apolipoprotein A-V gene, as described for the first time in 2001, is located proximal to the APOAI, APOCIII, and APOA-IV gene cluster on human 11q23 [39]. APOA5 encodes apolipoprotein (apo) AV, which is expressed in the liver and circulates on chylomicrons (CM), very low density lipoproteins (VLDL), and HDL. Common genetic variants of the apolipoprotein gene family members are related to variations in serum lipid levels.

Recently, a meta-analysis [40] showed that the APOA5 rs662799 C allele is associated with elevated circulating TG levels, regardless of ethnicity, indicating a possible mediating role for circulating TG in the association between the risk variant at APOA5 and the atherosclerotic process [41, 42]. This variant was correlated with not only higher plasma TG but also with lower HDL-C levels by our [22, 23] and other groups [38, 43]. Emerging data about APOA5-1131T > C suggested that APOA5 gene may have a direct effect above and beyond its effect on TG but, until now, needs to be confirmed by further studies [38]. In the Framingham Heart Study, an almost 2-fold increased risk of CVD was observed in females carrying the C allele of the −1131T > C [44]. Moreover, the relationship with IMT was observed for the rare allele of the −1131T > C SNP in overweight and obese subjects [45, 46].

Qiao et al. [47] observed that in type 2 diabetes patients, TG levels and the TG/HDL-C ratio were greater in those with TC and CC genotypes than in those with TT genotype subjects (*p* < 0.05). In addition, diabetic patients with CC genotype had greater carotid IMT than those with TT genotype (*p* = 0.080), although these data do not reach statistical significance.

We found a significant association between APOA5 CC genotype and cIMT (*p* = 0.045). Current evidence indicates that the increased risk of CVD is influenced by a merging of modifiable risk factors, ages, and/or genetics; however, the proportion of the contribution of these factors in modulating is still debated. Our findings suggest that even though the age of our study cohort was very young, women with C genotype probably experience the disadvantage of such genetic factor leading to CVD susceptibility in the form of a cIMT increase.

LDLR gene can regulate cholesterol metabolism. Among the several genetic variants identified at LDLR locus, the rs2228671 has been intensively studied and it has shown the strongest association with total and LDL-C levels across multiple populations [48–50] with the T allele being associated consistently, to a decreased risk of CAD [48]. Our previous findings are consistent with the previous literature; in fact, we showed that carriers of the rs2228671 T allele were significantly associated with the 3^rd^ trimester LDL-C levels in GDM women [22, 23].

Furthermore, we observed a significant association between CC APOA5/CC LDLR interaction and cIMT (*p* = 0.010). Surprisingly, women with CC genotype in APOA5 rs662799 in the absence of the T protective allele of LDLR rs2228671 present a cIMT increase. It would be interesting to further investigate the causal molecular mechanism underlying this interaction. We could hypothesize that the interaction effect of LDLR rs2228671 and APOA5 rs662799 implies a probable mitigation on cIMT values.

As expressed by Mecacci et al. [35], GDM can be considered as a “window into future health” in which it is advisable to adopt a healthy lifestyle to prevent or delay diabetes and/or CVD development postpartum.

In our previous study [23], we found that women with GDM had a greater BMI than the control group both in prepregnancy and at the end of pregnancy. In the present study, we analyzed women's lifestyle at follow-up: the pGDM women had a mean BMI that falls within the overweight range, as well as a high mean waist circumference (86.2 ± 16.1 cm). Moreover, no difference of adherence to the MedDiet, PA, and smoking was observed, although an inverse correlation between both the PREDIMED and the IPAQ scores with the cIMT values was found. This is an interesting feature supporting the potential role of preventive intervention. In fact, we noted that our cohort had a median MedDiet score of 7.5 (medium adherence), suggesting a poor adherence to healthy nutritional habits; also, a high percentage of them reported lower levels of PA. These modifiable risk factors can be easily acted upon to allow for early cardiovascular prevention.

In this view, tailored nutrition and lifestyle prescription represent a promising strategy for the prevention and management of metabolic syndrome [51]. In this regard, the main goals are a correct identification and stratification of GDM women at risk for CVD and an evaluation of the preventive strategies, as well as the improvement of postpartum screening.

Longer-term studies are indicated to define a potential role of lifestyle intervention [4]. Follow-up after GDM could be enhanced by similar quality and accountability measures requiring that patients and clinicians discuss future risks and referral to primary care as a standard of practice [36].

To our knowledge, this is the first study using multisectoral innovative biomarkers to evaluate the cardiometabolic risk in pGDM. Therefore, women with pGDM could be enrolled in follow-up programs designed to ensure continuous monitoring, thus providing effective prevention of both type 2 diabetes and CVD [7, 15].

It would be clinically valuable to have a risk marker during pregnancy so that long-term follow-ups and appropriate strategies of interventions can be focused on the women at greatest risk in a timely manner [52].

This study has some limitations. First of all, the sample size: this may have limited the statistical significance of metabolic and vascular function data. Second, non-pGDM women have not been involved. The present research is a preliminary small-scale study to evaluate the joint predictive role of lipid profile during pregnancy and of some genetic variants on cIMT taken as a parameter of subclinical atherosclerosis in a cohort of women with GDM history. Results obtained about the potential role of routine biomarkers and nutrigenetic variants in our small sample could be validated in a larger study.

However, our study provides a remarkable insight into the potential predictive role of both genetic factors and 3^rd^ trimester lipid profile for CVD susceptibility in pGDM. It will be crucial to replicate and expand our findings and further studies are warranted for better understanding of the potential gene-gene and gene-environment interactions, thus attributing a significant prognostic role to new and old biomarkers during pregnancy.

## Figures and Tables

**Table 1:** Characteristics of patients during pregnancy.

*Variable*	*N* = 28
Age (yr), *mean ± SD*	35.3 ± 3.8
School education, *n(%)*	
Low school	2 (7.1)
High school	14 (50.0)
University degree	12 (42.9)
Marital status, *n(%)*	
Single	5 (17.9)
Married	23 (82.1)
Employment, *n(%)*	
Employed	18 (64.3)
Unemployed	10 (35.7)
Prepregnancy BMI (kg/m^2^), *mean ± SD*	27.4 ± 7.1
BMI at OGTT (kg/m^2^), *mean ± SD*	30.8 ± 6.9
BMI at the end of pregnancy (kg/m^2^), *mean ± SD*	31.6 ± 5.8
Weight variation, *mean ± SD*	
OGTT vs prepregnancy (kg)	6.2 ± 4.7
Delivery vs prepregnancy (kg)	6.3 ± 5.9
Systolic blood pressure (mmHg), *mean ± SD*	113.0 ± 16.8
Diastolic blood pressure (mmHg), *mean ± SD*	72.2 ± 10.9
3^rd^ LDL-C (mg/dl), *mean ± SD*	150.5 ± 56.4
3^rd^ HDL-C (mg/dl), *mean ± SD*	68.8 ± 13.5
3^rd^ TC (mg/dl), *mean ± SD*	273.4 ± 51.0
3^rd^ TG (mg/dl), *mean ± SD*	242.7 ± 75.8
Smoking habit, *n(%)*	
No	20 (71.4)
Yes	2 (7.1)
Ex	6 (21.4)
OGTT, *mean ± SD*	
T0	94.5 ± 6.3
T60	161.8 ± 27.1
T120	134.6 ± 34.5
Fasting blood glucose (mg/dl), *mean ± SD*	83.0 ± 8.0
Family history of DM (1^st^ degree), *n(%)*	12 (42.9)
Previous GDM, *n(%)*	5 (17.9)

**Table 2:** Characteristics of patients at follow-up.

*Variable*	*N* = 28
Age (yr), *mean ± SD*	37.9 ± 4.2
Height (m), *mean ± SD*	1.60 ± 0.06
Weight (kg), *mean ± SD*	71.4 ± 20.7
BMI (kg/m^2^), *mean ± SD*	26.7 ± 9.2
Waist circumference (cm), *mean ± SD*	86.2 ± 16.1
Systolic blood pressure (mmHg), *mean ± SD*	118.5 ± 14.4
Diastolic blood pressure (mmHg), *mean ± SD*	74.8 ± 8.9
LDL-C (mg/dl), *mean ± SD*	110.1 ± 30.4
HDL-C (mg/dl), *mean ± SD*	54.2 ± 12.7
TC (mg/dl), *mean ± SD*	186.5 ± 33.2
TG (mg/dl), *mean ± SD*	110.8 ± 72.1
Fasting blood glucose (mg/dl), *mean ± SD*	91.4 ± 9.5
OGTT (mg/dl), *mean ± SD*	
T0	95.6 ± 9.4
T60	133.9 ± 35.7
T120	106.7 ± 24.4
HbA1C (mmol/mol), *mean ± SD*	35.4 ± 4.1
IPAQ, *n(%)*	
Low	14 (50.0)
Moderate	9 (32.1)
High	5 (17.9)
PREDIMED*, median (Q*_1_*-Q*_3_)	7.5 (6.0–9.0)
cIMT (mm), *mean ± SD*	0.51 ± 0.09
Homocisteina, *mean ± SD*	9.6 ± 3.1

**Table 3:** Genotypes distribution.

Genotype *n* (%)				No carrier	Carrier	HWE (*p* value)
TCF7L2	CC	CT	TT	CC	CT + TT	
rs7903146 (C > T)	8 (28.6)	10 (35.7)	10 (35.7)	8 (28.6)	20 (71.4)	*0.095*
PPARG2	CC	CG	GG	CC	CG + GG	
rs1801282 (C > G)	21 (75.0)	7 (25.0)	—	21 (75.0)	7 (25.0)	*0.389*
PPARGC1A	CC	CT	TT	CC	CT + TT	
rs8192678 (C > T)	9 (32.1)	16 (57.1)	3 (10.7)	9 (32.1)	19 (87.8)	*0.460*
APOA5	TT	CT	CC	TT	CT + CC	
rs662799 (T > C)	13 (46.4)	12 (42.9)	3 (10.7)	13 (46.4)	15 (53.6)	*0.857*
MC4R	TT	CT	CC	TT	CT + CC	
rs17782313 (T > C)	19 (67.9)	6 (21.4)	3 (10.7)	19 (67.9)	9 (32.1)	*0.099*
LDLR	CC	CT	TT	CC	CT + TT	
rs2228671 (C > T)	22 (78.6)	6 (21.4)	—	22 (78.6)	6 (21.4)	*0.460*
GCKR	CC	CT	TT	CC	CT + TT	
rs1260326 (C > T)	4 (14.3)	16 (57.1)	8 (28.6)	4 (14.3)	24 (85.7)	*0.293*
FTO	TT	TA	AA	TT	TA + AA	
rs9939609 (T > A)	8 (28.6)	10 (35.7)	10 (35.7)	8 (28.6)	20 (71.4)	*0.509*
MTHFR	CC	CT	TT	CC	CT + TT	
rs1801133 (C > T)	5 (17.9)	18 (64.3)	5 (17.9)	5 (17.9)	23 (82.1)	*0.190*

HWE = Hardy-Weinberg equilibrium.

## Data Availability

The data used to support the findings of this study are available from the corresponding author upon request.
